# The contribution of microbial biotechnology to mitigating coral reef degradation

**DOI:** 10.1111/1751-7915.12769

**Published:** 2017-07-11

**Authors:** Katarina Damjanovic, Linda L. Blackall, Nicole S. Webster, Madeleine J. H. van Oppen

**Affiliations:** ^1^ School of BioSciences The University of Melbourne Parkville Vic. 3010 Australia; ^2^ Australian Institute of Marine Science, PMB No 3 Townsville MC 4810 Qld Australia; ^3^ Australian Centre for Ecogenomics The University of Queensland Brisbane Qld 4072 Australia

## Abstract

The decline of coral reefs due to anthropogenic disturbances is having devastating impacts on biodiversity and ecosystem services. Here we highlight the potential and challenges of microbial manipulation strategies to enhance coral tolerance to stress and contribute to coral reef restoration and protection.

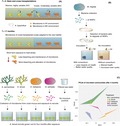

Reef‐forming scleractinian corals are foundation species of coral reefs, as they build the three‐dimensional structure of the reef, provide habitat to possibly millions of marine species and represent the most important primary producers through their association with microbial photosymbionts (*Symbiodinium* spp.) (Harrison and Booth, 2007). In addition to being extremely rich reservoirs of biodiversity, coral reefs greatly contribute to coastal protection, tourism and fisheries and their economic value is estimated in the range of billions of US dollars (Costanza *et al*., 1997; Burke *et al*., 2011). Coral reefs have suffered major declines over the past four decades, mainly due to anthropogenic disturbances acting on both local (e.g. overharvesting, pollution) and global (e.g. effects of climate change) scales (Bruno and Selig, 2007; Hoegh‐Guldberg, 2011; De'ath *et al*., 2012). Elevated sea surface temperature (SST) is a major driver of coral bleaching and mortality because it disrupts the critical relationship between corals and their endosymbiotic *Symbiodinium* (Hoegh‐Guldberg, 1999; Lesser, 2011). The recent global mass bleaching event that lasted from 2014 to 2016 and which caused unprecedented coral bleaching and mortality (Normile, 2016; Hughes *et al*., 2017) is reported to be the longest and most widespread on record (Cresset, 2016; NOAA Coral Reef Watch 2017).

Combatting the impacts of climate change and conserving marine resources are among the United Nations’ sustainable development goals (United Nations 2017). Despite increasing awareness of the threats of climate change to biodiversity and the establishment of guidelines to preserve marine ecosystems, environmental degradation is occurring faster than the pace of coral adaptation through natural selection on standing genetic variation (i.e. genetic adaptation) (Hoegh‐Guldberg, 2004; Hoegh‐Guldberg *et al*., 2007). Active interventions to help corals survive by augmenting their tolerance to and ability to recover from stress are therefore urgently required. Recently, the concept of assisted evolution has been proposed as a possible strategy for accelerating the rate of naturally occurring evolutionary processes and to develop corals capable of coping with current climate change trajectories (van Oppen *et al*., 2015). Assisted evolution includes selective breeding of coral, preconditioning of coral to sublethal stress, laboratory evolution of *Symbiodinium* followed by inoculation of the coral with tolerant algal symbionts, and manipulation of various members of the coral microbiome (van Oppen *et al*., 2015, 2017). This commentary focuses on the potential to mitigate coral reef degradation through the manipulation of coral‐associated prokaryotes.

Corals are colonized by a huge diversity of prokaryotes (Rohwer *et al*., 2002; Blackall *et al*., 2015), with distinct communities occupying various microhabitats within the host, including coral tissues, the surface mucus layer, the gastric cavity and the skeleton (Sweet *et al*., 2010; Bourne *et al*., 2016). Bacteria scavenge limiting nutrients (Knowlton and Rohwer, 2003; Zhang *et al*., 2015), deliver essential products to their host following carbon and nitrogen fixation (Lesser *et al*., 2007; Kimes *et al*., 2010) and participate in sulfur and phosphorus cycling (Raina *et al*., 2009; Zhang *et al*., 2015). Further, bacteria contribute to coral immune defences by occupying entry niches and by secreting antimicrobial peptides (Ritchie, 2006; Nissimov *et al*., 2009; Shnit‐Orland and Kushmaro, 2009). The composition of the coral microbiome can change with coral life stage, host health state, water temperature and acidity, nutrient levels, pollution, the presence of macroalgae, light intensity, depth or seasonal variation (Hernandez‐Agreda *et al*., 2016a; Glasl *et al*., 2017; Sweet and Bulling, 2017). Maintaining a healthy microbiome is thought to be essential for the well‐being of corals, as destabilization in the composition and function of their associated microbial communities has been shown to take place in diseased states (Frias‐Lopez *et al*., 2002; Jones *et al*., 2004; Gil‐Agudelo *et al*., 2006; Sato *et al*., 2013) and under stressful environmental conditions (Zaneveld *et al*., 2016). Elevated seawater temperatures and coral bleaching are typically associated with a shift towards opportunistic and/or pathogenic bacteria, with concomitant declines in coral health (Ritchie, 2006; Bourne *et al*., 2008; Littman *et al*., 2011; Lins‐de‐Barros *et al*., 2013; Tout *et al*., 2015). Conversely, in some instances coral‐associated microbes remain stable despite different host phenotypes (Hadaidi *et al*., 2017), changing environmental conditions (Teplitski *et al*., 2016) or in the presence of stressors (such as increased *p*CO_2_) (Webster *et al*., 2016; Zhou *et al*., 2016).

While an optimal microbiome may help protect the host from environmental pressures or compromised health by preserving beneficial functions, its dynamic nature may also confer an adaptive potential (Webster and Reusch, 2017). Whether by alteration in the relative abundance of certain species, acquisition of new species or variants from the environment or by mutations in the genomes of the existing community, modification of the microbiome is hypothesized to provide physiological flexibility to respond to environmental disturbances (Reshef *et al*., 2006; Rosenberg *et al*., 2007). For instance, reciprocal transplantation of *Acropora hyacinthus* fragments between thermally distinct environments on the same reef resulted in an adjustment of the microbial communities to the new conditions (Ziegler *et al*., 2017). While the microbiome of cross‐transplanted corals was indistinguishable from the microbiome of native corals in the same pool, it changed compared to the microbiome of the back‐transplanted counterparts (Fig. 1A). Moreover, when subjected to short‐term heat stress, corals that had spent the preceding 17 months in the more variable and warmer thermal regime were found to bleach less and retained a more stable microbiome. These results suggest that microbial community composition influences the response to heat stress, although host genetic adaptation and acclimatization are known to play additional roles (Barshis *et al*., 2013; Bay and Palumbi, 2014).

**Figure 1:**
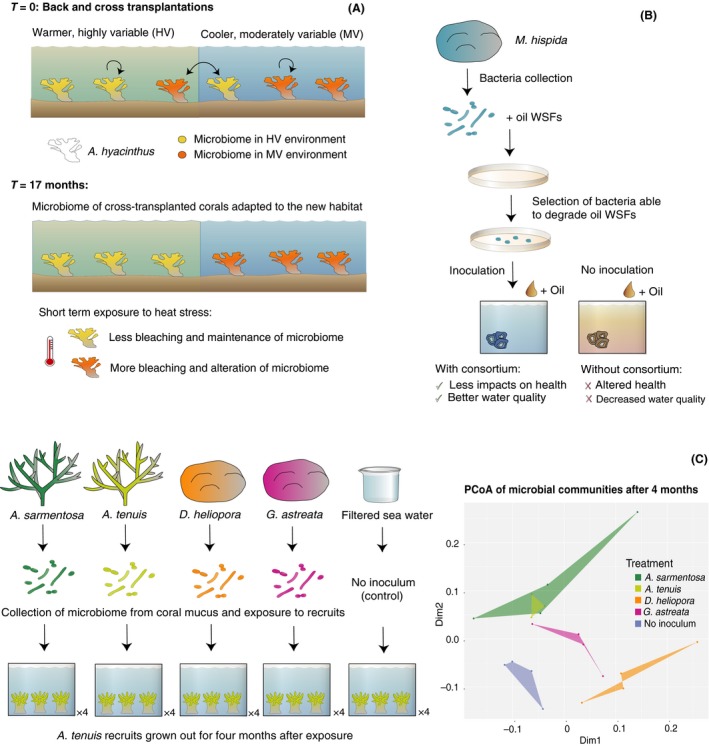
Examples of studies involving coral microbiome manipulation. A. Transplantation of *A. hyacinthus* fragments between regions of different thermal regimes induced a change in their microbiome (Ziegler *et al*., 2017). After 17 months, corals that inhabited the highly variable, warmer environment (HV) harboured a microbiome that was distinct from corals located in the cooler and more stable environment (MV). When exposed to short‐term heat stress, fragments from the HV environment bleached less, which could reflect a protective effect of their microbiome. B. Bacteria isolated from *M. hartii* were selected for their ability to degrade oil WSFs (dos Santos *et al*., 2015). Replicate coral colonies were inoculated with the selected bacterial consortium, while others were not exposed to these bacteria (controls). After subjecting the colonies to a treatment simulating an oil spill, the presence of bacteria able to degrade oil WSFs helped to preserve better water quality in the oil treatment and reduced negative effects on coral health. C. *A. tenuis* larvae from a common pool were distributed across 20 experimental tanks. Filtered sea water or 5‐μm filtered mucus collected from four different coral species were then introduced into four replicate tanks per treatment. Water flow was turned off overnight, and recruits were subsequently reared in flow‐through filtered sea water. After four months, recruits were sampled for 16S rRNA gene amplicon sequencing. PERMANOVA of the Bray–Curtis dissimilarities at the OTU level based on 97% sequence identity detected significant differences in the prokaryotic communities associated with recruits that were exposed to distinct inocula (pseudo *F*_4,14_ = 1.7015, *P* < 0.01).

As the composition and function of microbial communities seem to impact the fitness of their host, manipulation of resident prokaryotes could serve as a powerful tool to increase coral tolerance to stress and assist their adaptation to a changing environment. Such approaches are increasingly used in other biological systems. For instance, in humans, faecal microbiome transplantation is now accepted as an effective treatment for *Clostridium difficile* infections and is also gaining momentum for the treatment of other bowel conditions (Borody and Khoruts, 2012; Gupta *et al*., 2016). In agriculture, inoculation of rice plants with microbes collected from other plant species growing in extreme environments can enhance the rice plants’ tolerance to drought, salinity and low temperatures (Redman *et al*., 2011). In contrast to an entire microbiome transplant approach, bacterial species can also be selected and administered to the host to promote health. Probiotics are, for example, widely used in the aquaculture industry to stimulate growth, inhibit pathogens, improve water quality or augment tolerance to stress (Verschuere *et al*., 2000; Martinez Cruz *et al*., 2012; Boutin *et al*., 2013). While these approaches have not been readily applied in open marine systems, the biological control of coral diseases using phage therapy has already shown some promising outcomes in confined areas and in the laboratory for preventing and treating specific infections (Atad *et al*., 2012; Cohen *et al*., 2013).

Preliminary studies indicate that coral‐associated prokaryotes can be manipulated through inoculations with specific taxa. Bacteria collected from the coral *Mussismilia hartii* were cultured on a selective medium to isolate strains capable of degrading water‐soluble oil fractions (WSFs) (dos Santos *et al*., 2015). When subjected to conditions simulating an oil spill, polyps of *M. hartii* inoculated with a WSF‐degrading bacterial consortium (i.e. probiotic bacteria) were less negatively affected compared to the non‐inoculated polyps, as assessed by higher photosynthetic efficiencies of photosystem II of *Symbiodinium* (Fig. 1B). In this experiment, exposure to a specific microbial mixture therefore conferred health benefits to corals under environmental stress. Our preliminary experiments further support that the coral microbiome can be artificially influenced through microbiome inoculation (Data S1, S2, S3). Larvae of *Acropora tenuis* were exposed to the mucus collected from either *Acropora sarmentosa, Acropora tenuis, Diploastrea heliopora* or *Galaxea astreata*, and a no‐mucus treatment was included as a control (Fig. 1C). Mucus was chosen as the inoculum as it contains a high density of coral prokaryotes (Thompson *et al*., 2014) and can be easily collected after briefly exposing coral colonies to air (Brown and Bythell, 2005). Once settled, *A. tenuis* recruits were reared in filter‐sterilized and flow‐through sea water for 4 months, before being sampled to assess prokaryote microbiome composition. PERMANOVA of Bray–Curtis dissimilarities indicated that the microbiomes differed significantly across treatments suggesting that a single dosage drove the microbiome of experimental corals to develop in distinct directions (Fig. 1C).

Despite these recent encouraging outcomes from artificial microbial manipulations, important challenges for broadscale application in corals need to be addressed and overcome. First, the functions of the vast majority of coral‐associated prokaryotes are yet to be deciphered. We are still at a stage of correlating the presence of specific microbes with coral features as exemplified by *Endozoicomonas* (frequently reported to be present on healthy corals) (Kvennefors *et al*., 2010; Morrow *et al*., 2012; Meyer *et al*., 2014; Glasl *et al*., 2016; Neave *et al*., 2017), *Roseobacter* (particularly common in juvenile corals) (Apprill *et al*., 2012; Ceh *et al*., 2012, 2013; Sharp *et al*., 2012; Lema *et al*., 2014) and diazotrophs (nitrogen provisioning to corals) (Lema *et al*., 2012, 2015; dos Santos *et al*., 2014). A deeper knowledge of functions exerted by particular taxa will help designing optimal microbial inocula. Recently, ~200 distinct bacterial OTUs were able to be obtained in pure culture from the coral model *Exaiptasia pallida* using conventional methods (Röthig *et al*., 2016). These pure cultures represented *E. pallida*'s ‘key microbial associates’, and given the functional redundancy among members of the coral microbiome (Bell *et al*., 2005; Blackall *et al*., 2015; Sunagawa, 2015), these cultures provide opportunities for formulating a bacterial cocktail to evaluate their benefit to the host.

Genetic engineering should also be considered as an avenue to generate coral microbial inocula that possess desired characteristics. Such technologies have been applied to a wide range of organisms including bacteria, plants and mammals to study gene function or enhance phenotypic traits. In marine microbes, genetic engineering has been successfully employed to express high‐value bioactive compounds in the eukaryotic microalga, *Chlorella* (Yang *et al*., 2016), and for aquatic bioremediation and source of fuel in cyanobacteria (Lau *et al*., 2015). A framework has recently been proposed for creating transgenic *Symbiodinium*, which could ultimately lead to more stress‐tolerant variants (Levin *et al*., 2017). Likewise, coral‐associated bacteria could be transformed with genes of interest to produce strains that enhance the performance of the host under climate change. Genomes could for instance be edited at specific sites with the CRISPR‐Cas9 system (Hsu *et al*., 2014) or with mini‐Tn7 transposons (Lambertsen *et al*., 2004). The latter approach has already been used to label *Vibrio coralliilyticus* in corals and visualize host–pathogen interactions (Pollock *et al*., 2015). Developing genetically engineered symbionts could thus allow seeding vulnerable corals with organisms possessing proven beneficial properties.

Another challenge is the potential difficulty of manipulating microorganisms in open marine systems. While targeted microbiome transplants are performed in relatively closed systems by inoculating animal gut or soil, respectively, such precise interventions may prove less effective in the marine environment where the inoculum would dilute in sea water. Aquarium rearing would overcome this limitation by ensuring that a sufficiently high density of microorganisms reach corals. Propagation techniques of coral fragments and sexually derived propagules have considerably progressed over the last two decades for the purpose of coral reef restoration (Barton *et al*., 2015) and the *ex situ* rearing phase could theoretically be combined with microbiome inoculations.

A key uncertainty about the feasibility of manipulating microbes to enhance coral tolerance is whether the taxa in the inoculum will remain associated with the coral over time. The inherent variability of the microbiome (Escalante *et al*., 2015) may limit the utility of microbiome manipulation for sustainable development. Engineered microbiomes that are acquired environmentally, such as via the rhizosphere in the case of plants, are likely to require continuous selection for retention of the beneficial properties that are conferred to the host. However, members of the core microbiome may be more stably associated with their coral host, and this is a knowledge gap that needs to be urgently addressed. The coral core microbiome comprises bacterial taxa consistently associated with given coral species at a global scale despite contrasting environments (Ainsworth *et al*., 2015; Chu and Vollmer, 2016; Hernandez‐Agreda *et al*., 2016b). Such ubiquitous interactions across spatiotemporal boundaries suggest that these bacteria might perform critical functions for the host (reviewed in Hernandez‐Agreda *et al*. (2016a,b)). It has been reported that some members of the core microbiome are endosymbionts, residing both within host tissue (Ainsworth *et al*., 2006, 2015; Ainsworth and Hoegh‐Guldberg, 2009) and *Symbiodinium* cells (Ainsworth *et al*., 2015). We postulate that vertically inherited prokaryotes are more stably associated with the coral host compared to those that are horizontally acquired, as observed in other species such as the honeybee gut microbiome (Powell *et al*., 2014; Mueller and Sachs, 2015). The existence of conserved and intimate relationships between corals and microbes could open new avenues of research, where these stable communities would be targeted for manipulation.

Despite the recognition that coral reefs are threatened by human activities, measures to reduce negative impacts are insufficient. The United Nations have put forward targets that ought to be reached in the coming decade to protect marine ecosystems and avoid further adverse impacts (United Nations 2017). These goals include the sustainable management of marine zones by reducing pollution and destructive fishing, as well as the transfer of scientific knowledge to improve ocean health and support developing countries that rely on coral reefs. Intervention methods such as manipulation of the microbiome and genetic engineering have been successfully applied to terrestrial organisms to increase their tolerance to stress. Numerous lines of evidence suggest that a translation of these technologies to corals and their symbionts might effectively enhance coral resilience and contribute to the success of coral reef restoration efforts.

## Conflict of interest

None declared.

## Supporting information


**Fig. S1**. Relative abundance of phyla present in water (left) and coral (right) samples.
**Fig. S2.** PCoA plot of the Bray‐Curtis dissimilarities calculated at the phylum level depicts a striking difference between microbial communities present in coral and water samples.
**Fig. S3**. Box plots indicating beta diversity values in coral and water samples within each of the five inoculation treatments.

**Data S1**. Methods and results.
**Data S2**. Read counts for each sample at the genus level.

**Data S3**. Read counts for all samples at the phylum level.

## References

[mbt212769-bib-0001] Ainsworth, T.D. , and Hoegh‐Guldberg, O. (2009) Bacterial communities closely associated with coral tissues vary under experimental and natural reef conditions and thermal stress. Aquat Biol 4: 289–296.

[mbt212769-bib-0002] Ainsworth, T.D. , Fine, M. , Blackall, L.L. , and Hoegh‐Guldberg, O. (2006) Fluorescence in situ hybridization and spectral imaging of coral‐associated bacterial communities. Appl Environ Microbiol 72: 3016–3020. 16598010 10.1128/AEM.72.4.3016-3020.2006 PMC1449077

[mbt212769-bib-0003] Ainsworth, T.D. , Krause, L. , Bridge, T. , Torda, G. , Raina, J.B. , Zakrzewski, M. , *et al* (2015) The coral core microbiome identifies rare bacterial taxa as ubiquitous endosymbionts. ISME J 9: 2261–2274. 25885563 10.1038/ismej.2015.39 PMC4579478

[mbt212769-bib-0004] Apprill, A. , Marlow, H.Q. , Martindale, M.Q. , and Rappe, M.S. (2012) Specificity of associations between bacteria and the coral Pocillopora meandrina during early development. Appl Environ Microbiol 78: 7467–7475. 22904048 10.1128/AEM.01232-12 PMC3457095

[mbt212769-bib-0005] Atad, I. , Zvuloni, A. , Loya, Y. , and Rosenberg, E. (2012) Phage therapy of the white plague‐like disease of *Favia favus* in the Red Sea. Coral Reefs 31: 665–670.

[mbt212769-bib-0006] Barshis, D.J. , Ladner, J.T. , Oliver, T.A. , Seneca, F.O. , Traylor‐Knowles, N. , and Palumbi, S.R. (2013) Genomic basis for coral resilience to climate change. PNAS 110: 1387–1392. 23297204 10.1073/pnas.1210224110 PMC3557039

[mbt212769-bib-0007] Barton, J.A. , Willis, B.L. and Hutson, K.S. (2015) Coral propagation: a review of techniques for ornamental trade and reef restoration. Rev Aquacult [In press] doi: 10.1111/raq.12135.

[mbt212769-bib-0008] Bay, R.A. , and Palumbi, S.R. (2014) Multilocus adaptation associated with heat resistance in reef‐building corals. Curr Biol 24: 2952–2956. 25454780 10.1016/j.cub.2014.10.044

[mbt212769-bib-0009] Bell, T. , Newman, J.A. , Silverman, B.W. , Turner, S.L. , and Lilley, A.K. (2005) The contribution of species richness and composition to bacterial services. Nature 436: 1157–1160. 16121181 10.1038/nature03891

[mbt212769-bib-0010] Blackall, L.L. , B., W. and van Oppen, M.J. (2015) Coral – The world's most diverse symbiotic ecosystem. Mol Ecol 24: 5330–5347. 26414414 10.1111/mec.13400

[mbt212769-bib-0011] Borody, T.J. , and Khoruts, A. (2012) Fecal microbiota transplantation and emerging applications. Nat Rev Gastroenterol Hepatol 9: 88–96. 10.1038/nrgastro.2011.244 22183182

[mbt212769-bib-0012] Bourne, D. , Iida, Y. , Uthicke, S. , and Smith‐Keune, C. (2008) Changes in coral‐associated microbial communities during a bleaching event. ISME J 2: 350–363. 18059490 10.1038/ismej.2007.112

[mbt212769-bib-0013] Bourne, D.G. , Morrow, K.M. , and Webster, N.S. (2016) Insights into the coral microbiome: Underpinning the health and resilience of reef ecosystems. Annu Rev Microbiol 70: 317–340. 27482741 10.1146/annurev-micro-102215-095440

[mbt212769-bib-0014] Boutin, S. , Audet, C. , and Derome, N. (2013) Probiotic treatment by indigenous bacteria decreases mortality without disturbing the natural microbiota of Salvelinus fontinalis. Can J Microbiol 59: 662–670. 24102219 10.1139/cjm-2013-0443

[mbt212769-bib-0015] Brown, B.E. , and Bythell, J.C. (2005) Perspectives on mucus secretion in reef corals. Mar Ecol Prog Ser 296: 291–309.

[mbt212769-bib-0016] Bruno, J.F. , and Selig, E.R. (2007) Regional decline of coral cover in the Indo‐Pacific: timing, extent, and subregional comparisons. PLoS ONE 2: 1–8. 10.1371/journal.pone.0000711 PMC1933595 17684557

[mbt212769-bib-0017] Burke, L. , Reytar, K. and Spaulding, M.P.A. (2011). Reefs at Risk Revisited. Washington: World Resources Institute.

[mbt212769-bib-0018] Ceh, J. , Raina, J.B. , Soo, R.M. , van Keulen, M. , and Bourne, D.G. (2012) Coral‐bacterial communities before and after a coral mass spawning event on Ningaloo Reef. PLoS ONE 7: e36920. 22629343 10.1371/journal.pone.0036920 PMC3353996

[mbt212769-bib-0019] Ceh, J. , van Keulen, M. , and Bourne, D.G. (2013) Intergenerational transfer of specific bacteria in corals and possible implications for offspring fitness. Microb Ecol 65: 227–231. 22895828 10.1007/s00248-012-0105-z

[mbt212769-bib-0020] Chu, N.D. and Vollmer, S.V. (2016) Caribbean corals house shared and host‐specific microbial symbionts over time and space. Environ Microbiol Rep 8: 493–500. 27083502 10.1111/1758-2229.12412

[mbt212769-bib-0021] Cohen, Y. , Joseph Pollock, F. , Rosenberg, E. , and Bourne, D.G. (2013) Phage therapy treatment of the coral pathogen *Vibrio coralliilyticus* . Microbiologyopen 2: 64–74. 23239510 10.1002/mbo3.52 PMC3584214

[mbt212769-bib-0022] Costanza, R. , d'Arge, R. , de Groot, R. , Farber, S. , Grasso, M. , Hannon, B. , *et al* (1997) The value of the world's ecosystem services and natural capital. Nature 387: 253–260.

[mbt212769-bib-0023] Cresset, D. (2016) Coral crisis: Great Barrier Reef bleaching is “the worst we've ever seen. Nat News 1–2.

[mbt212769-bib-0024] De'ath, G. , Fabricius, K.E. , Sweatman, H. and Puotinen, M. (2012) The 27‐year decline of coral cover on the Great Barrier Reef and its causes. Proc Natl Acad Sci 109, 17995–17999. 23027961 10.1073/pnas.1208909109 PMC3497744

[mbt212769-bib-0025] Escalante, A.E. , Rebolleda‐Gomez, M. , Benitez, M. , and Travisano, M. (2015) Ecological perspectives on synthetic biology: insights from microbial population biology. Front Microbiol 6: 143. 25767468 10.3389/fmicb.2015.00143 PMC4341553

[mbt212769-bib-0026] Frias‐Lopez, J. , Zerkle, A.L. , Bonheyo, G.T. , and Fouke, B.W. (2002) Partitioning of Bacterial Communities between Seawater and Healthy, Black Band Diseased, and Dead Coral Surfaces. Appl Environ Microbiol 68: 2214–2228. 11976091 10.1128/AEM.68.5.2214-2228.2002 PMC127591

[mbt212769-bib-0027] Gil‐Agudelo, D.L. , Myers, C. , Smith, G.W. , and Kim, K. (2006) Changes in the microbial communities associated with *Gorgonia ventalina* during aspergillosis infection. Dis Aquat Organ 69: 89–94. 16703770 10.3354/dao069089

[mbt212769-bib-0028] Glasl, B. , Herndl, G.J. and Frade, P.R. (2016) The microbiome of coral surface mucus has a key role in mediating holobiont health and survival upon disturbance. ISME J 10: 2280–2292. 26953605 10.1038/ismej.2016.9 PMC4989324

[mbt212769-bib-0029] Glasl, B. , Webster, N.S. , and Bourne, D.G. (2017) Microbial indicators as a diagnostic tool for assessing water quality and climate stress in coral reef ecosystems. Mar Biol 164: 1–18.27980349

[mbt212769-bib-0030] Gupta, S. , Allen‐Vercoe, E. , and Petrof, E.O. (2016) Fecal microbiota transplantation: in perspective. Ther Adv Gastroenterol 9: 229–239. 10.1177/1756283X15607414 PMC4749851 26929784

[mbt212769-bib-0031] Hadaidi, G. , Rothig, T. , Yum, L.K. , Ziegler, M. , Arif, C. , Roder, C. , *et al* (2017) Stable mucus‐associated bacterial communities in bleached and healthy corals of *Porites lobata* from the Arabian Seas. Sci Rep 7: 45362. 28361923 10.1038/srep45362 PMC5374439

[mbt212769-bib-0032] Harrison, P. L. and Booth, D. J. (2007). Coral reefs: naturally dynamic and increasingly disturbed ecosystems In Marine Ecology. Connell S. D., Gillanders B. M. (eds.). Melbourne: Oxford University Press, pp. 316–377.

[mbt212769-bib-0033] Hernandez‐Agreda, A. , Gates, R.D. and Ainsworth, T.D. (2016a) Defining the core microbiome in corals’ microbial soup. Trends Microbiol 25: 125–140. 27919551 10.1016/j.tim.2016.11.003

[mbt212769-bib-0034] Hernandez‐Agreda, A. , Leggat, W. , Bongaerts, P. , and Ainsworth, T.D. (2016b) The microbial signature provides insight into the mechanistic basis of coral success across reef habitats. MBio 7: e00560–16. 27460792 10.1128/mBio.00560-16 PMC4981706

[mbt212769-bib-0035] Hoegh‐Guldberg, O. (1999) Climate change, coral bleaching and the future of the world's coral reefs. Mar Freshwater Res 50: 839–866.

[mbt212769-bib-0036] Hoegh‐Guldberg, O. (2004) Coral reefs in a century of rapid environmental change. Symbiosis 37: 1–31.

[mbt212769-bib-0037] Hoegh‐Guldberg, O. (2011). The Impact of Climate Change on Coral Reef Ecosystems In Coral Reefs: An Ecosystem in Transition. Dubinsky Z. and Stambler N. (eds.). Netherlands: Springer, pp. 391–403.

[mbt212769-bib-0038] Hoegh‐Guldberg, O. , Mumby, P.J. , Hooten, A.J. , Steneck, R.S. , Greenfield, P. , Gomez, E. , *et al* (2007) Coral Reefs Under Rapid Climate Change and Ocean Acidification. Science 318: 1737–1742. 18079392 10.1126/science.1152509

[mbt212769-bib-0039] Hsu, P.D. , Lander, E.S. , and Zhang, F. (2014) Development and applications of CRISPR‐Cas9 for genome engineering. Cell 157: 1262–1278. 24906146 10.1016/j.cell.2014.05.010 PMC4343198

[mbt212769-bib-0040] Hughes, T.P. , Kerry, J.T. , Alvarez‐Noriega, M. , Alvarez‐Romero, J.G. , Anderson, K.D. , Baird, A.H. , *et al* (2017) Global warming and recurrent mass bleaching of corals. Nature 543: 373–377. 28300113 10.1038/nature21707

[mbt212769-bib-0041] Jones, R.J. , Browyer, J. , Hoegh‐Guldberg, O. , and Blackall, L.L. (2004) Dynamics of a temperature‐related coral disease outbreak. Mar Ecol Prog Ser 281: 63–77.

[mbt212769-bib-0042] Kimes, N.E. , Van Nostrand, J.D. , Weil, E. , Zhou, J. , and Morris, P.J. (2010) Microbial functional structure of *Montastraea faveolata*, an important Caribbean reef‐building coral, differs between healthy and yellow‐band diseased colonies. Environ Microbiol 12: 541–556. 19958382 10.1111/j.1462-2920.2009.02113.x

[mbt212769-bib-0043] Knowlton, N. , and Rohwer, F. (2003) Multispecies microbial mutualisms on coral reefs: the host as a habitat. Am Nat 162: 51–62. 10.1086/378684 14583857

[mbt212769-bib-0044] Kvennefors, E.C.E. , Sampayo, E. , Ridgway, T. , Barnes, A.C. , and Hoegh‐Guldberg, O. (2010) Bacterial Communities of Two Ubiquitous Great Barrier Reef Corals Reveals Both Site‐ and Species‐Specificity of Common Bacterial Associates. PLoS ONE 5: 1–14. 10.1371/journal.pone.0010401 PMC2861602 20454460

[mbt212769-bib-0045] Lambertsen, L. , Sternberg, C. , and Molin, S. (2004) Mini‐Tn7 transposons for site‐specific tagging of bacteria with fluorescent proteins. Environ Microbiol 6: 726–732. 15186351 10.1111/j.1462-2920.2004.00605.x

[mbt212769-bib-0046] Lau, N.S. , Matsui, M. , and Abdullah, A.A. (2015) Cyanobacteria: photoautotrophic microbial factories for the sustainable synthesis of industrial products. Biomed Res Int 2015: 754934. 26199945 10.1155/2015/754934 PMC4496466

[mbt212769-bib-0047] Lema, K.A. , Willis, B.L. , and Bourne, D.G. (2012) Corals form characteristic associations with symbiotic nitrogen‐fixing bacteria. Appl Environ Microbiol 78: 3136–3144. 22344646 10.1128/AEM.07800-11 PMC3346485

[mbt212769-bib-0048] Lema, K.A. , Bourne, D.G. , and Willis, B.L. (2014) Onset and establishment of diazotrophs and other bacterial associates in the early life history stages of the coral Acropora millepora. Mol Ecol 23: 4682–4695. 25156176 10.1111/mec.12899

[mbt212769-bib-0049] Lema, K.A. , Clode, P.L. , Kilburn, M.R. , Thornton, R. , Willis, B.L. and Bourne, D.G. (2015) Imaging the uptake of nitrogen‐fixing bacteria into larvae of the coral Acropora millepora. ISME J 10: 1804–1808. 26696324 10.1038/ismej.2015.229 PMC4918436

[mbt212769-bib-0050] Lesser, M.P. (2011). Coral Bleaching: Causes and Mechanisms In Coral Reefs: An Ecosystem in Transition. Dubinsky Z. and Stambler N. (eds.). Netherlands: Springer, pp. 405–419.

[mbt212769-bib-0051] Lesser, M.P. , Falcón, L.I. , Rodríguez‐Román, A. , Enríquez, S. , Hoegh‐Guldberg, O. , and Iglesias‐Prieto, R. (2007) Nitrogen fixation by symbiotic cyanobacteria provides a source of nitrogen for the scleractinian coral *Montastraea cavernosa* . Mar Ecol Prog Ser 346: 143–152.

[mbt212769-bib-0052] Levin, R.A. , Voolstra, C.R. , Agrawal, S. , Steinberg, P.D. , Suggett, D. and van Oppen, M. (2017) Engineering strategies to decode and enhance coral symbiont biology. Front Microbiol [In press] doi: 10.3389/fmicb.2017.01220. 10.3389/fmicb.2017.01220 PMC5492045 28713348

[mbt212769-bib-0053] Lins‐de‐Barros, M.M. , Cardoso, A.M. , Silveira, C.B. , Lima, J.L. , Clementino, M.M. , Martins, O.B. , *et al* (2013) Microbial community compositional shifts in bleached colonies of the Brazilian reef‐building coral *Siderastrea stellata* . Microb Ecol 65: 205–213. 22864853 10.1007/s00248-012-0095-x

[mbt212769-bib-0054] Littman, R. , Willis, B.L. , and Bourne, D.G. (2011) Metagenomic analysis of the coral holobiont during a natural bleaching event on the Great Barrier Reef. Environ Microbiol Rep 3: 651–660. 23761353 10.1111/j.1758-2229.2010.00234.x

[mbt212769-bib-0055] Martinez Cruz, P. , Ibanez, A.L. , Monroy Hermosillo, O.A. , and Ramirez Saad, H.C. (2012) Use of probiotics in aquaculture. ISRN Microbiol 2012: 916845. 23762761 10.5402/2012/916845 PMC3671701

[mbt212769-bib-0056] Meyer, J.L. , Paul, V.J. , and Teplitski, M. (2014) Community shifts in the surface microbiomes of the coral Porites astreoides with unusual lesions. PLoS ONE 9: e100316. 24937478 10.1371/journal.pone.0100316 PMC4061089

[mbt212769-bib-0057] Morrow, K.M. , Moss, A.G. , Chadwick, N.E. , and Liles, M.R. (2012) Bacterial associates of two Caribbean coral species reveal species‐specific distribution and geographic variability. Appl Environ Microbiol 78: 6438–6449. 22773636 10.1128/AEM.01162-12 PMC3426691

[mbt212769-bib-0058] Mueller, U.G. , and Sachs, J.L. (2015) Engineering microbiomes to improve plant and animal health. Trends Microbiol 23: 606–617. 26422463 10.1016/j.tim.2015.07.009

[mbt212769-bib-0059] Neave, M.J. , Michell, C.T. , Apprill, A. , and Voolstra, C.R. (2017) Endozoicomonas genomes reveal functional adaptation and plasticity in bacterial strains symbiotically associated with diverse marine hosts. Sci Rep 7: 40579. 28094347 10.1038/srep40579 PMC5240137

[mbt212769-bib-0060] Nissimov, J. , Rosenberg, E. , and Munn, C.B. (2009) Antimicrobial properties of resident coral mucus bacteria of *Oculina patagonica* . FEMS Microbiol Lett 292: 210–215. 19191871 10.1111/j.1574-6968.2009.01490.x

[mbt212769-bib-0061] NOAA Coral Reef Watch . (2017). “Global coral bleaching 2014‐2017: Status and an appeal for observations.” URl https://coralreefwatch.noaa.gov/satellite/analyses_guidance/global_coral_bleaching_2014-17_status.php. Accessed 01 May 2017

[mbt212769-bib-0062] Normile, D. (2016). “Massive bleaching killed 35% of the coral on the northern end of the Great Barrier Reef.” URL http://www.sciencemag.org/news/2016/05/massive-bleaching-killed-35-coral-northern-end-great-barrier-reef. Accessed 01 May 2017

[mbt212769-bib-0063] van Oppen, M.J.H. , Oliver, J.K. , Putnam, H.M. and Gates, R.D. (2015) Building coral reef resilience through assisted evolution. Proc Natl Acad Sci 112: 2307–2313. 25646461 10.1073/pnas.1422301112 PMC4345611

[mbt212769-bib-0064] van Oppen, M.J. , Gates, R.D. , Blackall, L.L. , Cantin, N. , Chakravarti, L.J. , Chan, W.Y. , *et al* (2017) Shifting paradigms in restoration of the world's coral reefs. Glob Chang Biol [In press] DOI 10.1111/gcb.13647. 10.1111/gcb.13647 28247459

[mbt212769-bib-0065] Pollock, F.J. , Krediet, C.J. , Garren, M. , Stocker, R. , Winn, K. , Wilson, B. , *et al* (2015) Visualization of coral host–pathogen interactions using a stable GFP‐labeled *Vibrio coralliilyticus* strain. Coral Reefs 34: 655–662.

[mbt212769-bib-0066] Powell, J.E. , Martinson, V.G. , Urban‐Mead, K. , and Moran, N.A. (2014) Routes of acquisition of the gut microbiota of the honey bee *Apis mellifera* . Appl Environ Microbiol 80: 7378–7387. 25239900 10.1128/AEM.01861-14 PMC4249178

[mbt212769-bib-0067] Raina, J.B. , Tapiolas, D. , Willis, B.L. , and Bourne, D.G. (2009) Coral‐associated bacteria and their role in the biogeochemical cycling of sulfur. Appl Environ Microbiol 75: 3492–3501. 19346350 10.1128/AEM.02567-08 PMC2687302

[mbt212769-bib-0068] Redman, R.S. , Kim, Y.O. , Woodward, C.J.D.A. , Greer, C. , Espino, L. , Doty, L.S. , and Rodriguez, R.J. (2011) Increased fitness of rice plants to abiotic stress via habitat adapted symbiosis: a strategy for mitigating impacts of climate change. PLoS ONE 6: 1–10. 10.1371/journal.pone.0014823 PMC3130040 21750695

[mbt212769-bib-0069] Reshef, L. , Koren, O. , Loya, Y. , Zilber‐Rosenberg, I. , and Rosenberg, E. (2006) The coral probiotic hypothesis. Environ Microbiol 8: 2068–2073. 17107548 10.1111/j.1462-2920.2006.01148.x

[mbt212769-bib-0070] Ritchie, K.B. (2006) Regulation of microbial populations by coral surface mucus and mucus‐associated bacteria. Mar Ecol Prog Ser 322: 1–14.

[mbt212769-bib-0071] Rohwer, F. , Seguritan, V. , Azam, F. , and Knowlton, N. (2002) Diversity and distribution of coral‐associated bacteria. Mar Ecol Prog Ser 243: 1–10.

[mbt212769-bib-0072] Rosenberg, E. , Koren, O. , Reshef, L. , Efrony, R. , and Zilber‐Rosenberg, I. (2007) The role of microorganisms in coral health, disease and evolution. Nat Rev Microbiol 5: 355–362. 17384666 10.1038/nrmicro1635

[mbt212769-bib-0073] Röthig, T. , Costa, R.M. , Simona, F. , Baumgarten, S. , Torres, A.F. , Radhakrishnan, A. , *et al* (2016) Distinct bacterial communities associated with the coral model *Aiptasia* in aposymbiotic and symbiotic states with Symbiodinium. Front. Mar. Sci. 3: 1–12.

[mbt212769-bib-0074] dos Santos, H.F. , Carmo, F.L. , Duarte, G. , Dini‐Andreote, F. , Castro, C.B. , Rosado, A.S. , *et al* (2014) Climate change affects key nitrogen‐fixing bacterial populations on coral reefs. ISME J 8: 2272–2279. 24830827 10.1038/ismej.2014.70 PMC4992079

[mbt212769-bib-0075] dos Santos, H.F. , Duarte, G.A. , Rachid, C.T. , Chaloub, R.M. , Calderon, E.N. , Marangoni, L.F. , *et al* (2015) Impact of oil spills on coral reefs can be reduced by bioremediation using probiotic microbiota. Sci Rep 5, 18268. 26658023 10.1038/srep18268 PMC4677405

[mbt212769-bib-0076] Sato, Y. , Willis, B.L. and Bourne, D.G. (2013) Pyrosequencing‐based profiling of archaeal and bacterial 16S rRNA genes identifies a novel archaeon associated with black band disease in corals. Environ Microbiol 15: 2994–3007. 24112537 10.1111/1462-2920.12256

[mbt212769-bib-0077] Sharp, K.H. , Distel, D. , and Paul, V.J. (2012) Diversity and dynamics of bacterial communities in early life stages of the Caribbean coral Porites astreoides. ISME J 6: 790–801. 22113375 10.1038/ismej.2011.144 PMC3309355

[mbt212769-bib-0078] Shnit‐Orland, M. , and Kushmaro, A. (2009) Coral mucus‐associated bacteria: a possible first line of defense. FEMS Microbiol Ecol 67: 371–380. 19161430 10.1111/j.1574-6941.2008.00644.x

[mbt212769-bib-0079] Sunagawa, S. (2015) Structure and function of the global ocean microbiome. Science 348, 1261359. 1261351‐1261359‐1261359. 25999513 10.1126/science.1261359

[mbt212769-bib-0080] Sweet, M.J. and Bulling, M.T. (2017) On the importance of the microbiome and pathobiome in coral health and disease. Front Mar Sci 4: 1–11.

[mbt212769-bib-0081] Sweet, M.J. , Croquer, A. , and Bythell, J.C. (2010) Bacterial assemblages differ between compartments within the coral holobiont. Coral Reefs 30: 39–52.

[mbt212769-bib-0082] Teplitski, M. , Krediet, C.J. , Meyer, J.L. and Ritchie, K.B. (2016) Microbial interactions on coral surfaces and within the coral holobiont. In The Cnidaria, Past, Present and Future. Goffredo S. and Dubinsky Z. (Eds.). Springer International Publishing: Switzerland, pp. 331–346.

[mbt212769-bib-0083] Thompson, J.R. , Rivera, H.E. , Closek, C.J. , and Medina, M. (2014) Microbes in the coral holobiont: partners through evolution, development, and ecological interactions. Front Cell Infect Microbiol 4: 176. 25621279 10.3389/fcimb.2014.00176 PMC4286716

[mbt212769-bib-0084] Tout, J. , Siboni, N. , Messer, L.F. , Garren, M. , Stocker, R. , Webster, N.S. , *et al* (2015) Increased seawater temperature increases the abundance and alters the structure of natural *Vibrio* populations associated with the coral *Pocillopora damicornis* . Front Microbiol 6: 432. 26042096 10.3389/fmicb.2015.00432 PMC4435422

[mbt212769-bib-0085] United Nations . (2017). “Sustainable Development Goals.” URL https://sustainabledevelopment.un.org/sdgs. Accessed 15 May 2017.

[mbt212769-bib-0086] Verschuere, L. , Rombaut, G. , Sorgeloos, P. , and Verstraete, W. (2000) Probiotic bacteria as biological control agents in aquaculture. Microbiol Mol Biol Rev 64: 655–671. 11104813 10.1128/mmbr.64.4.655-671.2000 PMC99008

[mbt212769-bib-0087] Webster, N.S. and Reusch, T. (2017) Microbial contributions to the persistence of coral reefs. ISME J [Epub ahead of print] doi: 10.1038/ismej.2017.66. 10.1038/ismej.2017.66 PMC5607359 28509908

[mbt212769-bib-0088] Webster, N.S. , Negri, A.P. , Botté, E.S. , Laffy, P.W. , Flores, F. , Noonan, S. , *et al* (2016) Host‐associated coral reef microbes respond to the cumulative pressures of ocean warming and ocean acidification. Sci Rep 6: 19324. 26758800 10.1038/srep19324 PMC4725835

[mbt212769-bib-0089] Yang, B. , Liu, J. , Jiang, Y. , and Chen, F. (2016) *Chlorella* species as hosts for genetic engineering and expression of heterologous proteins: progress, challenge and perspective. Biotechnol J 11: 1244–1261. 27465356 10.1002/biot.201500617

[mbt212769-bib-0090] Zaneveld, J.R. , Burkepile, D.E. , Shantz, A.A. , Pritchard, C.E. , McMinds, R. , Payet, J.P. , *et al* (2016) Overfishing and nutrient pollution interact with temperature to disrupt coral reefs down to microbial scales. Nat Commun 7: 11833. 27270557 10.1038/ncomms11833 PMC4899628

[mbt212769-bib-0091] Zhang, Y. , Ling, J. , Yang, Q. , Wen, C. , Yan, Q. , Sun, H. , *et al* (2015) The functional gene composition and metabolic potential of coral‐associated microbial communities. Sci Rep 5: 16191. 26536917 10.1038/srep16191 PMC4633650

[mbt212769-bib-0092] Zhou, G. , Yuan, T. , Cai, L. , Zhang, W. , Tian, R. , Tong, H. , *et al* (2016) Changes in microbial communities, photosynthesis and calcification of the coral *Acropora gemmifera* in response to ocean acidification. Sci Rep 6: 1–9. 27786309 10.1038/srep35971 PMC5082368

[mbt212769-bib-0093] Ziegler, M. , Seneca, F.O. , Yum, L.K. , Palumbi, S.R. , and Voolstra, C.R. (2017) Bacterial community dynamics are linked to patterns of coral heat tolerance. Nat Commun 8: 14213. 28186132 10.1038/ncomms14213 PMC5309854

