# Supplementary material for: The contribution of microbial biotechnology to mitigating coral reef degradation
Source: Microb Biotechnol. 2017 Jul 11;10(5):1236–43. doi: 10.1111/1751-7915.12769 (PMC5609283; doi:10.1111/1751-7915.12769)
Supplement: Supplementary file 1 — Fig. S1. Relative abundance of phyla present in water (left) and coral (right) samples. Fig. S2. PCoA plot of the Bray‐Curtis dissimilarities calculated at the phylum level depicts a striking difference between microbial communities present in coral and water samples. Fig. S3. Box plots indicating beta diversity values in coral and water samples within each of the five inoculation treatments. [file MBT2-10-1236-s001.docx]

**Supporting Information**

**Methods**

*Coral spawning*

Colonies of the coral *Acropora tenuis* were collected from Trunk Reef (S 18°18.505 E 146°51.978), Australia, and transported to the Australian Institute of Marine Science (AIMS) on the 22^nd^ of November 2015. The corals were deployed in 3000 L tanks with running seawater. On the night of spawning (30 November 2015), single colonies were isolated in 50 L plastic bins filled with 0.4 µm filtered seawater and gamete bundles from five colonies were collected from the water surface. Eggs and sperm were washed, separated, and the sperm from all colonies was combined in a bucket. The eggs were then mixed with the sperm and the gametes left to fertilize. After 3 hr, developing embryos were transferred into 500 L tanks containing 0.4 µm filtered flow-through seawater and a low level of aeration. Four days after spawning, the larvae were distributed to 20 replicate 60 L tanks containing 0.4 µm filtered and UV-treated flow-through seawater at a density of approximately 10 larvae/mL. The tanks were kept at 27°C and under a 12 hr light / 12 hr dark illumination cycle reaching a maximum light intensity of 120 µmol·m^-2^·s^-1^ after 5 hr ramping. The bottom of each tank was covered with autoclaved terracotta tiles and autoclaved crustose coralline algae chips were spread over the tiles to induce settlement. The larvae were exposed to mucus collected from adult coral colonies 2 hr after they were put in the experimental tanks.

*Exposure of recruits to coral mucus*

Fragments from two colonies of *Acropora tenuis*, *Acropora sarmentosa*, *Galaxea astreata* and *Diploastrea heliopora*, also collected from Trunk reef, were used as mucus donors to inoculate the larvae. The corals were exposed to air for 15 min and placed underwater for 30 min in one 50 L plastic bin per species. The released mucus was collected with a plastic pipette and 50 mL were transferred to sterile conical polypropylene tubes. The mucus samples were diluted 40 times before being filtered through a 5 µm syringe filter; dilution of the mucus was necessary to avoid clogging of the filters. A volume of 0.5 L of the filtrate obtained from each of the four coral species was distributed among four replicate tanks already containing the larvae. The same volume of filtered seawater was poured in another four tanks as a “no inoculum” control treatment. Water flow and pumps were turned off overnight after inoculation.

*Rearing of recruits and sampling*

At one week and again at two weeks post-settlement, *A. tenuis* recruits were inoculated with a clonal pure culture of *Symbiodinium* C1. The same amount of *Symbiodinium* was added to each tank at 1 pm; following inoculation, the lights, water flow and pumps were turned off during 4 hr. The recruits were fed twice daily with the *Shellfish Diet 1800 Instant Algae* microalga mix at a concentration of 5000 cells/mL and the tanks were frequently cleaned with acrylic scrubber pads to remove algal films developing on the surfaces. After six weeks, small snails were introduced to all tanks to help reduce growth of filamentous algae on the tiles. Recruits were regularly sampled and counted until they reached the age of four months (31^st^ March). Results of this last sampling time-point are reported here. Using sterile scalpel blades, about 30 polyps were scraped off from the tiles of each tank, placed in cryovials, and snap-frozen in liquid nitrogen. One litre of water was also collected from each tank and 0.22-µm filtered with a peristaltic pump to analyse prokaryotic communities present in the water column surrounding the recruits.

*DNA extraction and amplification*

DNA was extracted from the samples following slight modifications of the protocol reported in Wilson et al. 2002. Briefly, the recruits (weighing about 25 mg) were placed in 1.5 mL sterile microcentrifuge tubes containing 250 mL of extraction buffer. Six µL of lysozyme at 10 mg/mL were added to all tubes, which were subsequently incubated on ice for 15 min. About 40 mg of sterile glass beads and 6 µL of Proteinase K at 20 mg/mL were then added to the tubes. The samples were bead-beaten at 4 m/s for 20 sec and incubated at 65°C for 2 hr. After this step, 62.5 µL of KOAc at 5 M were pipetted into the tubes and the latter were incubated on ice for 30 min. After spinning the tubes at 25,000 x *g* for 15 min at room temperature, the supernatant was transferred into new 1.5 mL sterile microcentrifuge tubes and 0.8 vol. isopropanol was added. The solutions were left at room temperature for 15 min and centrifuged again at 25,000 x *g* for 15 min. After removing the supernatant, the precipitate was washed with 100 µl of 70% ethanol, centrifuged at 25,000 *g* for 3 min, air-dried and resuspended overnight in 20 µL MilliQ water.

The hypervariable region V4 of the 16S rRNA gene was amplified using revised versions of the primers 515F [5’-TCGTCGGCAGCGTCAGATGTGTATAAGAGACAGGTGYCAGCMGCCGCGGTAA-3’] ([Apprill et al. 2015](#_ENREF_1)) and 806R [5’-GTCTCGTGGGCTCGGAGATGTGTATAAGAGACAGGGACTACNVGGGTWTCTAAT-3’] ([Parada et al. 2015](#_ENREF_8)). The underlined segments represent Illumina adapters (Illumina, San Diego, CA, USA). The PCRs were conducted in triplicate 10 µL reactions using the Qiagen Multiplex Mastermix and 0.2 µM of each primer. The amplification cycles were set as following: 95°C for 15 min; 28 cycles each at 94°C for 30 sec, 57°C for 1 min, 72°C for 30 sec; a final extension at 72°C for 10 min. The PCR products were then pooled and sent to Ramaciotti Centre for Genomics (UNSW, Sydney) for library preparation and sequencing on the Illumina MiSeq system.

*Bioinformatics*

Amplicon sequences were processed using the QIIME pipeline v2.7.9 ([Caporaso et al. 2010](#_ENREF_2)). Poor quality and short sequences (< 170 bp) were removed using Trimmomatic. The module PEAR v0.9.6 ([Zhang et al. 2014](#_ENREF_18)) was used to assemble reads, allowing to merge overlapping paired-end sequences and produce the full length 16S V4 fragments. After generating fasta files and adding sample identifiers to the header of each sequence, sequences were checked against the “RDP Gold” chimeric-free database ([UCHIME Accessed 2016](#_ENREF_15)) to delete chimeras. Sequences were clustered using uclust ([Edgar 2010](#_ENREF_3)) at 97% sequence similarity level and OTUs were assigned with the open reference picking method against the SILVA 119 rRNA database ([Quast et al. 2013](#_ENREF_10)). QIIME was subsequently used to filter out chloroplast sequences, remove OTUs representing very low abundance (<0.005%) and generate the final OTU table.

Statistical analyses were performed in R v3.3.2 ([R Development Core Team 2008](#_ENREF_11)). Dissimilarity between samples subjected to the five different treatments was visualised with Principal Coordinate Analysis using the cmdscale function in the R package Vegan ([Oksanen et al. 2016](#_ENREF_7)). Differences between the microbial communities were tested by PERMANOVA of Bray-Curtis dissimilarities using the Adonis function. Beta diversity was calculated with the function betadisper and the homogeneity of variances verified with the function permutest.

**Additional results**

*Coral-associated microbial communities are different from the ones in surrounding seawater*

Already at the phylum level, microbial community composition associated with coral recruits is different from the community present in the surrounding seawater. Fig. S1 shows the relative abundance of all phyla detected in water and coral samples.

|  |  |
| --- | --- |
| **Figure S1**. Relative abundance of phyla present in water (left) and coral (right) samples. Sample names refer to treatment (Acr: *A. sarmentosa*; Aten: *A. tenuis*; Dip: *D. heliopora*; Gal: *G. astreata*; No: no inoculum) and type (H2O: water sample). Letters A-D indicate the four replicate tanks of one treatment. | |

While Archaea are abundant in water samples, they do not seem closely associated with recruits. Other works have also reported that Archaea tend to establish only loose associations with corals ([Frade et al. 2016](#_ENREF_4)).

PERMANOVA of the Bray-Curtis dissimilarities at the phylum level confirmed that microbial communities collected from water and coral samples were significantly different (pseudo F_(1,28)_ = 278.785, p < 0.01). Principal coordinate analysis of the Bray-Curtis dissimilarities allows to visualise the divergence between microbial communities present in coral and water samples (Fig. S2). Such observations are congruent with many previous studies describing different microbial communities between the coral host and surrounding seawater ([Wegley et al. 2007](#_ENREF_16), [Sunagawa et al. 2010](#_ENREF_12), [Tremblay et al. 2010](#_ENREF_14), [Sweet et al. 2011](#_ENREF_13), [Lema et al. 2014](#_ENREF_5), [Li et al. 2014](#_ENREF_6), [Pratte et al. 2015](#_ENREF_9), [Frade et al. 2016](#_ENREF_4)). This suggests that corals are able to select for particular microbial partners from the environment and thus actively shape their microbiome.

| 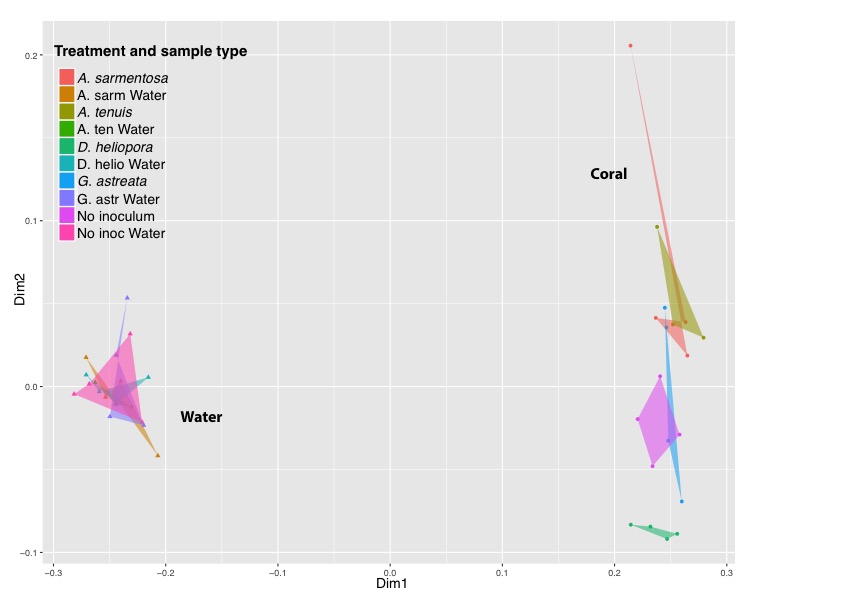 |
| --- |
| **Figure S2.** PCoA plot of the Bray-Curtis dissimilarities calculated at the phylum level depicts a striking difference between microbial communities present in coral and water samples. |

*Coral and water samples differ in beta-diversity*

Beta diversity was calculated to assess the variability in microbial communities at the genus level in coral and water samples. There was no statistically significant difference in beta diversity between the five treatments within coral and within water samples. However, there was a significantly higher variation in coral samples as compared to water samples (F_(1,36)_ = 90.941, p < 0.01). Microbial communities therefore seem to vary more from coral to coral than between different water samples (Fig. S3).

|  |
| --- |
| **Figure S3** Box plots indicating beta diversity values in coral and water samples within each of the five inoculation treatments. |

References

Apprill, A., S. McNally, R. Parsons and L. Weber (2015). "Minor revision to V4 region SSU rRNA 806R gene primer greatly increases detection of SAR11 bacterioplankton." Aquatic Microbial Ecology **75**(2): 129-137.

Caporaso, J. G., J. Kuczynski, J. Stombaugh, K. Bittinger, F. D. Bushman, E. K. Costello, N. Fierer, A. Gonzalez Pena, J. K. Goodrich, J. I. Gordon, G. A. Huttley, S. Kelley, D. Knights, J. E. Koening, R. E. Ley, C. A. Lozupone, D. McDonald, B. D. Muegge, M. Pirrung, J. Reeder, J. R. Sevinsky, P. J. Turnbaugh, W. A. Walters, J. Widmann, T. Yatsunenko, J. Zaneveld and R. Knight (2010). "QIIME allows analysis of high- throughput community sequencing data." Nature methods **7**(5): 335-336.

Edgar, R. C. (2010). "Search and clustering orders of magnitude faster than BLAST." Bioinformatics **26**(19): 2460-2461.

Frade, P. R., K. Roll, K. Bergauer and G. J. Herndl (2016). "Archaeal and Bacterial Communities Associated with the Surface Mucus of Caribbean Corals Differ in Their Degree of Host Specificity and Community Turnover Over Reefs." PLoS One **11**(1): e0144702.

Lema, K. A., D. G. Bourne and B. L. Willis (2014). "Onset and establishment of diazotrophs and other bacterial associates in the early life history stages of the coral Acropora millepora." Mol Ecol **23**(19): 4682-4695.

Li, J., Q. Chen, L. J. Long, J. D. Dong, J. Yang and S. Zhang (2014). "Bacterial dynamics within the mucus, tissue and skeleton of the coral Porites lutea during different seasons." Sci Rep **4**: 7320.

Oksanen, J., F. G. Blanchet, M. Friendly, R. Kindt, P. Legendre, D. McGlinn, P. R. Minchin, R. B. O'Hara, C. L. Simpson, P. Solymos, M. Henry, H. Stevens, E. Szoecs and H. Wagner (2016). "vegan: Community Ecology Package. R package version 2.4-1."

Parada, A. E., D. M. Needham and J. A. Fuhrman (2015). "Every base matters: assessing small subunit rRNA primers for marine microbiomes with mock communities, time series and global field samples." Environ Microbiol.

Pratte, Z. A., L. L. Richardson and D. K. Mills (2015). "Microbiota shifts in the surface mucopolysaccharide layer of corals transferred from natural to aquaria settings." J Invertebr Pathol **125**: 42-44.

Quast, C., E. Pruesse, P. Yilmaz, J. Gerken, T. Schweer, P. Yarza, J. Peplies and F. O. Glockner (2013). "The SILVA ribosomal RNA gene database project: improved data processing and web-based tools." Nucleic Acids Res **41**(Database issue): D590-596.

R Development Core Team. (2008). "R: A language and environment for statistical computing. ." 2016, from <http://www.R-project.org>.

Sunagawa, S., C. M. Woodley and M. Medina (2010). "Threatened corals provide underexplored microbial habitats." PloS one **5**(3): 1-7.

Sweet, M. J., A. Croquer and J. C. Bythell (2011). "Dynamics of bacterial community development in the reef coral Acropora muricata following experimental antibiotic treatment." Coral Reefs **30**(4): 1121-1133.

Tremblay, P., M. G. Weinbauer, C. Rottier, Y. Guérardel, C. Nozais and C. Ferrier-Pagès (2010). "Mucus composition and bacterial communities associated with the tissue and skeleton of three scleractinian corals maintained under culture conditions." Journal of the Marine Biological Association of the United Kingdom **91**(03): 649-657.

UCHIME. (Accessed 2016). "Downloads." from <http://drive5.com/uchime/uchime_download.html>.

Wegley, L., R. Edwards, B. Rodriguez-Brito, H. Liu and F. Rohwer (2007). "Metagenomic analysis of the microbial community associated with the coral Porites astreoides." Environmental Microbiology **9**(11): 2707-2719.

Wilson, K. J., Y. Li, V. Whan, S. A. Lehnert, K. Byrne, S. S. Moore, S. Pongsomboon, A. Tassanakajon, G. Rosenberg, E. Ballment, Z. Fayazi, J. Swan, M. J. Kenway and J. A. H. Benzie (2002). "Genetic mapping of the black tiger shrimp Penaeus monodon with Amplified Fragment Length Polymorphisms." Aquaculture **204**: 297-309.

Zhang, J., K. Kobert, T. Flouri and A. Stamatakis (2014). "PEAR: a fast and accurate Illumina Paired-End reAd mergeR." Bioinformatics **30**(5): 614-620.
